# Accessory Genomes Drive Independent Spread of Carbapenem-Resistant Klebsiella pneumoniae Clonal Groups 258 and 307 in Houston, TX

**DOI:** 10.1128/mbio.00497-22

**Published:** 2022-03-31

**Authors:** William C. Shropshire, An Q. Dinh, Michelle Earley, Lauren Komarow, Diana Panesso, Kirsten Rydell, Sara I. Gómez-Villegas, Hongyu Miao, Carol Hill, Liang Chen, Robin Patel, Bettina C. Fries, Lilian Abbo, Eric Cober, Sara Revolinski, Courtney L. Luterbach, Henry Chambers, Vance G. Fowler, Robert A. Bonomo, Samuel A. Shelburne, Barry N. Kreiswirth, David van Duin, Blake M. Hanson, Cesar A. Arias

**Affiliations:** a Center for Infectious Diseases, School of Public Health, University of Texas Health Science Center, Houston, Texas, USA; b Center for Antimicrobial Resistance and Microbial Genomics, Division of Infectious Diseases, University of Texas Health Science Center at Houston McGovern Medical School, Houston, Texas, USA; c Division of Infectious Diseases, Houston Methodist Hospital, Houston, Texas, USA; d Center for Infectious Diseases Research, Houston Methodist Research Institute, Houston, Texas, USA; e The Biostatistics Center, The George Washington University, Rockville, Maryland, USA; f Department of Biostatistics and Data Science, School of Public Health, University of Texas Health Science Center, Houston, Texas, USA; g Duke Clinical Research Institute, Duke University Medical Center, Durham, North Carolina, USA; h Center for Discovery and Innovation, Hackensack Meridian Health, Nutley, New Jersey, USA; i Division of Clinical Microbiology, Department of Laboratory Medicine and Pathology, Mayo Clinic, Rochester, Minnesota, USA; j Division of Infectious Diseases, Department of Medicine, Mayo Clinic, Rochester, Minnesota, USA; k Department of Medicine, Infectious Disease Division, Stony Brook University, Stony Brook, New York, USA; l Veteran’s Administration Medical Center, Northport, New York, USA; m Division of Infectious Diseases, Department of Medicine, University of Miami Miller School of Medicine and Jackson Health System, Miami, Florida, USA; n Department of Infectious Diseases, Cleveland Clinic, Cleveland, Ohio, USA; o School of Pharmacy, Medical College of Wisconsin, Milwaukee, Wisconsin, USA; p Division of Infectious Diseases, University of North Carolina at Chapel Hill, Raleigh, North Carolina, USA; q Department of Medicine, University of California San Francisco, San Francisco, USA; r Division of Infectious Diseases, Duke University, Durham, North Carolina, USA; s Department of Pharmacology, Case Western Reserve University School of Medicine, Cleveland, Ohio, USA; t Department of Molecular Biology and Microbiology, Case Western Reserve University School of Medicine, Cleveland, Ohio, USA; u Department of Biochemistry, Case Western Reserve University School of Medicine, Cleveland, Ohio, USA; v Department of Proteomics and Bioinformatics, Case Western Reserve University School of Medicine, Cleveland, Ohio, USA; w CWRU-Cleveland VAMC Center for Antimicrobial Resistance and Epidemiology, Cleveland, Ohio, USA; x Department of Infectious Diseases, The University of Texas MD Anderson Cancer Center, Houston, Texas, USA; y Department of Genomic Medicine, The University of Texas MD Anderson Cancer Center, Houston, Texas, USA; McMaster University

**Keywords:** CG258, CG307, carbapenem-resistant *Klebsiella pneumoniae*, divergent evolution, genomic surveillance, mobile genetic elements

## Abstract

Carbapenem-resistant Klebsiella pneumoniae (CR*Kp*) is an urgent public health threat. Worldwide dissemination of CR*Kp* has been largely attributed to clonal group (CG) 258. However, recent evidence indicates the global emergence of a CR*Kp* CG307 lineage. Houston, TX, is the first large city in the United States with detected cocirculation of both CR*Kp* CG307 and CG258. We sought to characterize the genomic and clinical factors contributing to the parallel endemic spread of CG258 and CG307. CR*Kp* isolates were collected as part of the prospective, Consortium on Resistance against Carbapenems in Klebsiella and other *Enterobacterales* 2 (CRACKLE-2) study. Hybrid short-read and long-read genome assemblies were generated from 119 CR*Kp* isolates (95 originated from Houston hospitals). A comprehensive characterization of phylogenies, gene transfer, and plasmid content with pan-genome analysis was performed on all CR*Kp* isolates. Plasmid mating experiments were performed with CG307 and CG258 isolates of interest. Dissection of the accessory genomes suggested independent evolution and limited horizontal gene transfer between CG307 and CG258 lineages. CG307 contained a diverse repertoire of mobile genetic elements, which were shared with other non-CG258 K. pneumoniae isolates. Three unique clades of Houston CG307 isolates clustered distinctly from other global CG307 isolates, indicating potential selective adaptation of particular CG307 lineages to their respective geographical niches. CG307 strains were often isolated from the urine of hospitalized patients, likely serving as important reservoirs for genes encoding carbapenemases and extended-spectrum β-lactamases. Our findings suggest parallel cocirculation of high-risk lineages with potentially divergent evolution.

## INTRODUCTION

Carbapenem-resistant Klebsiella pneumoniae (CR*Kp*) causes significant worldwide morbidity and mortality and is of paramount concern in nosocomial settings (1). Since the identification of K. pneumoniae isolates harboring the carbapenem-hydrolyzing enzyme K. pneumoniae carbapenemase (KPC) in 1996, global dissemination of CR*Kp* has occurred (2). CR*Kp* now accounts for the majority of carbapenem-resistant *Enterobacterales* (CRE) infections in the United States (3, 4). Infections caused by CR*Kp* are difficult to treat since these isolates often harbor resistance to multiple antibiotics, limiting therapeutic options (5).

Global dissemination of CR*Kp* has been largely attributed to the clonal expansion of a genetic lineage of K. pneumoniae designated clonal group 258 (CG258), first identified in the United States in 2008 (6–8). Spread of CG258 has been associated with carriage of genes encoding the KPC enzyme (6, 9), most notably on F-type pKpQIL plasmids (10). More recently, another CR*Kp* genetic lineage, designated CG307, has emerged and appears to be spreading in countries such as Italy, Pakistan, Colombia, and the United States (11–13). Isolates in the K. pneumoniae CG307 lineage carry distinct genomic features that may confer virulence and colonization advantages, such as F-type plasmid-borne glycogen synthesis gene clusters and urea transport systems (12). These potential virulence determinants are present in conjunction with established pathogenic factors, such as the *mrk* gene cluster (encoding a type 3 pili), and extracellular polysaccharides (capsule and lipopolysaccharides) found across all multidrug-resistant (MDR) K. pneumoniae lineages (14). Carriage of *bla*_KPC_ on pKpQIL and N-type plasmids in CG307 isolates has been documented (12), however, not at the same prevalence as CG258 (15, 16). There is a strong association of the CG307 lineage with the carriage of *bla*_CTX-M-15_, a gene encoding an extended-spectrum β-lactamase (ESBL) (11). Of interest, a molecular dating analysis estimated that CG307 arose in 1994 (11) near the estimated year of CG258 emergence (6), suggesting that these two high-risk genetic lineages have evolved in parallel.

A recent large U.S. cohort study of patients infected or colonized with CRE (Consortium on Resistance against Carbapenems in Klebsiella and other *Enterobacterales* 2 [CRACKLE-2] study) identified K. pneumoniae CG307 as the second most common lineage of CR*Kp* after CG258 (7% versus 64% out of 593 isolates, respectively) (3). Of note, in that study, the majority (72%) of the CG307 K. pneumoniae isolates were found in the Houston, TX, metropolitan region. This observation is in agreement with previous reports describing the molecular epidemiology of multidrug-resistant K. pneumoniae in a hospital network in Houston, TX (17). Furthermore, the CRACKLE-2 results suggested that Houston had become the first large city in the United States with high endemicity of CR*Kp* detected where K. pneumoniae CG307 and CG258 appear to be cocirculating and undergoing parallel expansion. Nevertheless, the molecular epidemiology of these two predominant lineages cocirculating in the same geographical locale, in particular, the extent to which they share adaptive traits found in their accessory genome, remains poorly understood.

To dissect the factors that drove the parallel cocirculation of CG307 and CG258, as well as the high endemicity of CR*Kp* in the Houston area, we generated complete assemblies of K. pneumoniae *sensu stricto* recovered from patients enrolled in the CRACKLE-2 cohort in Houston and other sites in the United States. We assessed the potential intra- and interclade transmission of vectors responsible for carbapenem resistance between CG258 and CG307 and their respective correlated gene content and characterized clinical outcome differences in patients colonized or infected with CR*Kp*.

## RESULTS

### Patients with CG307 colonization or infection may have lower 30-day mortality than those with CG258.

Clinical epidemiological features stratified by CG for the Houston, TX, isolates are presented in Table 1. The crude 30-day mortality for the full Houston cohort (*n* = 73) was 17.8%; (13/73 [95% confidence interval (CI), 7.8 to 22.6%]). When comparing clinical features across the CG258 and CG307 groups, patients with CG258 exhibited a statistically significantly higher 30-day mortality than those infected/colonized with CG307, albeit with a small number of events. Patients with CG307 infection/colonization exhibited a higher proportion of samples isolated from urine (65.6%) than those with CG258 (37.0%), but this association did not reach statistical significance (*P* = 0.068). Conversely, patients with CG258 infection/colonization had a higher proportion of isolates from blood (14.8% versus 0%) and respiratory cultures (25.9% versus 12.5%) than the CG307 patient group.

**TABLE 1:** Clinical features and outcomes of CG258 versus CG307 from Houston, TX

Feature	Clonal Group		*P* value^*a*^
	CG258 (*n* = 27)	CG307 (*n* = 32)	
Age (yr) (SD)	60.7 (17.8)	63.4 (18.3)	0.72
Male (%)	14 (52)	11 (34)	0.20
Ethnicity			0.24%
Hispanic or Latino	6 (22)	4 (13)	
Not Hispanic or Latino	18 (67)	27 (84)	
Unknown	2 (7)	0 (0)	
Not reported	1 (4)	1 (3)	
Origin (%)			0.75
Home/transfer outside U.S.	20 (74)	24 (75)	
Long-term acute-care/hospital transfer	1 (4)	0 (0)	
Long-term-care hospice	6 (22)	8 (25)	
Charlson score (SD)	2.6 (1.9)	3.6 (2.5)	0.13
Pitt score (SD)	2.6 (2.9)	1.7 (1.9)	0.30
Renal dysfunction (yes) (%)	7 (26)	15 (47)	0.11
Culture source			0.068%
Blood	4 (15)	0 (0)	
Respiratory	7 (26)	4 13)	
Urine	10 (37)	21 (66)	
Wound	3 (11)	4 (13)	
Other	3 (11)	3 (9)	
Infection (yes) (%)	6 (22)	9 (28)	0.77
Hospital duration (days) (SD)	13.9 (14.8)	10.8 (7.9)	0.96
30-day mortality (%)	8 (30)	1 (3)	0.008

a Continuous outcomes tested with Wilcoxon rank sum test; Categorical outcomes tested with Fisher’s exact test.

### Molecular epidemiological features of CG258 and CG307 circulating in the same geographical area reveal distinct virulence and antimicrobial resistance gene content.

**FIG 1:**
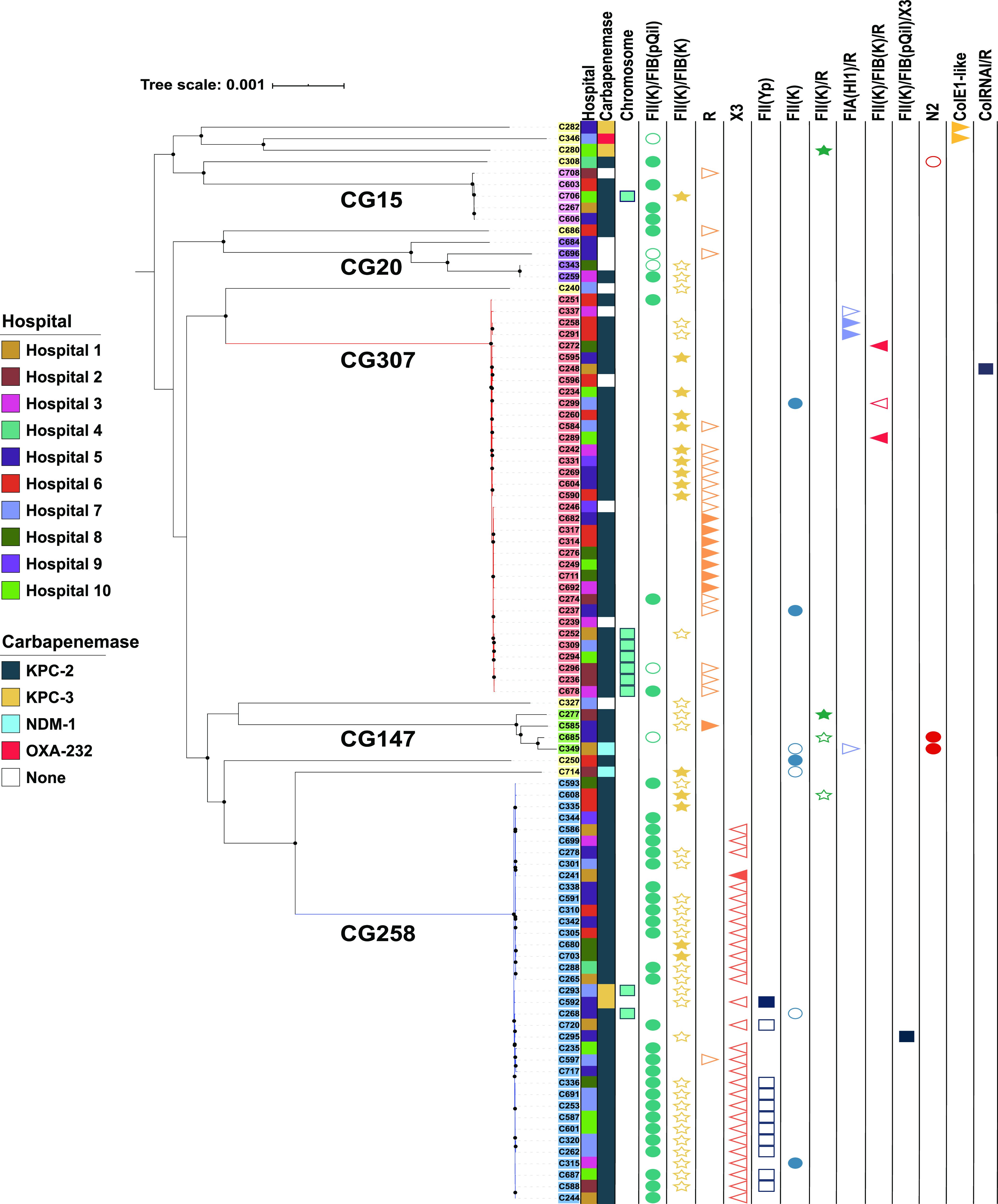
Core gene pangenomic population structure of 94 CR*Kp* isolates in the Houston, TX, cohort. Maximum-likelihood phylogenetic, midpoint rooted tree demonstrating the two predominant cocirculating clades, CG258 (blue clade) and CG307 (red clade). Internal node bootstrap values of ≥95% are denoted as black circles. Label backgrounds indicate the 6 hierarchical clustering groups identified using Bayesian analysis of population structure. Clonal groups associated with each hierarchical group are as follows: CG258 (blue), CG307 (red), CG147 (green), CG15 (pink), CG20 (purple), and other CGs (yellow). The first column indicates each of the 10 hospital sites in Houston, TX. The second column indicates carbapenemase carriage. The third column indicates if carbapenemase carriage was chromosomal. The final 13 columns indicate the plasmid vectors that were identified to have at least one sample with carbapenemase carriage. Carbapenemase carriage of each plasmid vector is distinguished by carbapenemase positive (filled shape) and carbapenemase negative (empty shape).

Most isolates identified as CR*Kp* belonged to the taxon K. pneumoniae *sensu stricto* (94/95; 98.9%), except for one isolate (C719, identified as K. quasipneumoniae subsp. *quasipneumoniae*). The predominant CR*Kp* sequence types identified in the Houston cohort were CG258 (37/95; 38.9%) and CG307 (35/95; 36.8%). The remaining isolates belonged to CG15 (*n* = 5), CG20 (*n* = 4), CG147 (*n* = 4), and a mixture of other sequence types (*n* = 10) (Fig. 1). There were no observed correlations between clonal group and hospital location across the Houston metropolitan area (Fisher exact *P* value = 0.8), suggesting each site had a similar distribution to the overall circulation of sequence types in this hospital system (Fig. S1 at https://gitlab.com/carmig_dissertations/shropshire_dissertation/crkp_supplemental_files).

Molecular epidemiological features of the Houston K. pneumoniae isolates are shown in Table 2. The median genome size of isolates belonging to CG307 was smaller than that of CG258 and other heterogeneous sequence types, although there were no significant differences in chromosome sizes between the three CGs. The smaller genome size of CG307 was due to a smaller number of plasmids compared to CG258 or other CGs (Table 2). The mean number of acquired antimicrobial resistance (AMR) genes per genome, i.e., the average number of nonintrinsic AMR genes found per CR*Kp* isolate, was 7.8 ± 5.0 genes (Fig. S2A at https://gitlab.com/carmig_dissertations/shropshire_dissertation/crkp_supplemental_files). The median number of acquired AMR genes stratified by group was similar between CG258 (5; interquartile range [IQR] = 6), CG307 (7; IQR = 5), and other CGs (9; IQR = 10.5). We did not find a statistically significant difference across these subsets in the numbers of genes encoding AMR determinants of different classes (i.e., number of antibiotic classes with at least one resistance determinant per genome) (Fig. S2B). We also analyzed AMR and virulence determinants using the Kleborate composite resistance scoring metric (18) along with the categorical virulence score (see Materials and Methods). We found no statistically significant difference in Kleborate resistance scores between CG258 and CG307 (adjusted [adj.] *P* value = 0.3). However, CG258 isolates had a statistically significantly higher resistance score than the group of other heterogenous sequence types (adj. *P* value = 0.007). The Kleborate composite virulence scores in Table 1 reflect that the Houston CG307 isolates lacked genes encoding acquired/nonintrinsic siderophores (e.g., yersiniabactin, salmochelin, aerobactin), the genotoxin colibactin gene cluster, and hypermucoidity genes (*rmpA* and *rmpA2*), which are commonly found virulence determinants in hypervirulent lineages of K. pneumoniae *sensu stricto* (19, 20).

**TABLE 2:** Pangenomic features of K. pneumoniae^*a*^

Feature	Clonal Group:			*P* value^*b*^
	CG258 (*n* = 37)	CG307 (*n* = 35)	Other CG (*n* = 23)	
Genome size(bp) (IQR)	5,695,223 (321,076)	5,551,257 (142,518)	5,666,705 (119,114)	0.0001
Chromosome size(bp) (IQR)	5,322,183 (187,234)	5,357,994 (15,382)	5,302,444 (155,756)	0.3
GC content (%) (IQR)	57.1 (0.2)	57.3 (0.1)	57.2 (0.2)	<0.0001
No. of coding genes (IQR)	5,380 (269)	5,158 (139)	5,286 (120)	<0.0001
No. of plasmids (IQR)	4 (2)	2 (1)	4 (3)	<0.0001
No. of resistance genes (IQR)	5 (6)	7 (5)	9 (11)	0.3
No. of resistance classes (IQR)	6 (3)	6 (1)	6 (7)	0.3
Resistance score (SD)^*c*^	2.1 (0.2)	1.9 (0.4)	1.6 (0.8)	0.01
Virulence score (SD)^*c*^	0.7 (1.0)	0 (0.0)	0.7 (1.4)	0.0006

a CG, clonal group; IQR, interquartile range; median values are reported with IQR; means are reported with SD.

b Kruskal-Wallis rank sum test *P* value.

c Composite antimicrobial resistance and virulence scores based on scoring schema developed by the Holt et al. (21) Kleborate tool (https://github.com/katholt/Kleborate).

Multiple conjugative elements (ICE*Kp*) carrying virulence factors integrated in the chromosome of K. pneumoniae lineages have been previously described (19). These mobile genetic elements largely contribute to hypervirulent, high-risk strains of K. pneumoniae commonly observed in Asian countries (5). There were 35.1% (13/37) of CG258 Houston, TX, isolates that carried a chromosomally inserted ICE*Kp* with an associated yersiniabactin gene (*ybt*) cluster. The ICE*Kp* in most of these CG258 isolates harbored an ICE*Kp*10-*ybt*17 sequence type except for one Houston CG258 isolate (C592) that carried a novel *ybt* sequence type. All Houston CG258 isolates harboring ICE*Kp* also contained the ICE*Kp*-associated colibactin gene cluster from the *clb*3 lineage. This is consistent with multiple, unique ICE*Kp* chromosomal insertions within the CG258 clade having been previously observed in other CG258 backgrounds found in other geographical regions (19). In contrast, all Houston CG307 isolates lacked ICE*Kp* integration. Only one CG307 isolate in the non-Houston isolates (C4693 recovered from a patient in Georgia) harbored a chromosomal ICE*Kp*, which belongs to the ICE*Kp*12 lineage and encodes the *ybt*16 sequence type. An important genomic epidemiological observation relating to the population structure of K. pneumoniae is the rare convergence of multidrug-resistant (MDR) and hypervirulence genetic determinants (5, 21). We identified two CR*Kp* isolates with predicted resistance to >3 antimicrobial classes, C308 (ST23) and C346 (ST231), that harbored aerobactin-encoding *iuc* genes along with *bla*_KPC-2_ and *bla*_OXA-232_, respectively, suggesting that the convergence of MDR and hypervirulence genetic determinants is occurring in our study population.

### The population structure in cocirculating CG258 and CG307 indicates nested subgroups within each lineage.

The pangenome of the full cohort (*n* = 121), using 94 CR*Kp* Houston isolates with 12 CG307 and 12 CG258 isolates collected from other CRACKLE-2 U.S. hospital sites (*n* = 48 sites) plus 3 references (see Materials and Methods), consisted of 13,049 genes, of which 3,908 (29.9%) made up the core genome, defined as gene groups included in ≥99% of the full cohort. The overall species median nucleotide divergence, a measure of genetic variation within a population based on normalized polymorphism counts, was 0.6% (median pairwise single-nucleotide polymorphism [SNP] difference = 22,417 SNPs), suggesting that less than 1% of the core genome nucleotide sites were variant sites. This nucleotide diversity is comparable to previously shown measures of genetic variation within the K. pneumoniae *sensu stricto* phylogroup based on core gene alignment (21).

**FIG 2:**
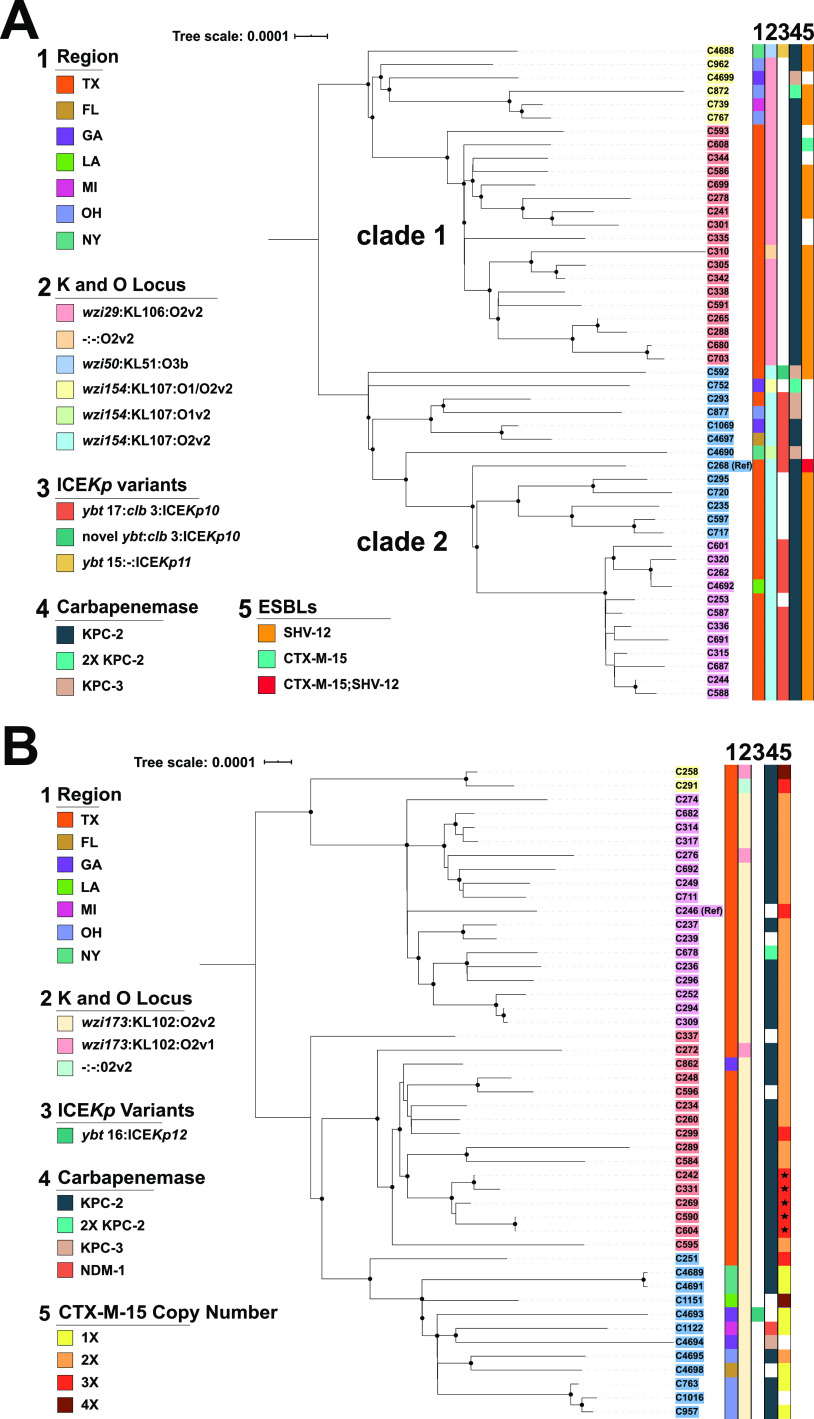
Core SNP-inferred Maximum Likelihood (ML) phylogenomic trees of CG258 and CG307 isolates using a midpoint rooted tree. Internal node bootstrap values of ≥95% are denoted as black circles. Isolate label backgrounds indicate hierarchical clustering groups identified using Bayesian analysis of population structure for each respective sublineage. Legend designations are as follows: (1) region from which isolate was collected, (2) capsular synthesis/lipopolysaccharide (LPS) allele type, (3) ICE*Kp* variant with yersiniabactin and colibactin gene cluster lineages listed, (4) carbapenemase carriage status, (5) ESBL carriage (CG258)/*bla*_CTX-M-15_ copy number (CG307). (A) CG258 core SNP phylogeny using a reference-based alignment with a C268 isolate. (B) CG307 core SNP phylogeny using reference-based alignment with a C246 isolate. Stars in the *bla*_CTX-M-15_ copy number column (i.e., column 5) indicate an isolate with one truncated *bla*_CTX-M-15_ copy.

To dissect lineage-specific population structures, recombination-free, reference-based core SNP maximum-likelihood phylogenetic trees were created for both CG258 and CG307 (Fig. 2). The intergroup median nucleotide divergence of CG258 and CG307 was 0.6% (median pairwise SNP difference = 23,304 SNPs), which was comparable to overall species divergence. The intragroup median nucleotide divergence calculated for the CG258 group was 0.013% (median pairwise SNP difference = 508 SNPs), a core genome nucleotide diversity expected for a clonal group showing a more homogenous core genome at the clonal group level, compared to the overall species level. We identified two clades within the CG258 lineage (Fig. 2A), split largely by the capsule synthesis locus (*cps*), as previously described (7, 22). Clade 1 (*wzi 29*/KL106) and clade 2 (*wzi 154*/KL107) encompassed 45% and 51% of CG258 K. pneumoniae isolates, respectively. The remaining 4% of CG258 isolates had unique capsular synthesis loci and/or a large IS*Kpn26*-mediated deletion in this region (a region known to have a high rate of recombination). There were nested population structures within each clade that segregated by geographical site (Houston versus other U.S. CRACKLE-2 sites), with a clearer delineation within clade 1. There was a strong association of isolates harboring ICE*Kp10* (primarily the *ybt*17 lineage) with clade 2 isolates (18/25; 72%) not observed in clade 1 isolates (Fisher exact *P* value = 5e-6).

The CG307 group (Fig. 2B) was less divergent than CG258, with a median nucleotide divergence of 0.004% (median pairwise SNP difference = 145 SNPs). There was a marked geographical split of the CG307 group, correlating with the predicted four hierarchical clusters within the core population structure. A majority of CG307 isolates had the same K and O loci (*wzi173* allele associated with the KL102 locus and the O2v1 [3/47] or O2v2 [33/47] loci, respectively), with the exception of one isolate (C291) which had a 28,813-bp IS*Kpn26* associated deletion in the *cps* locus.

**FIG 3:**
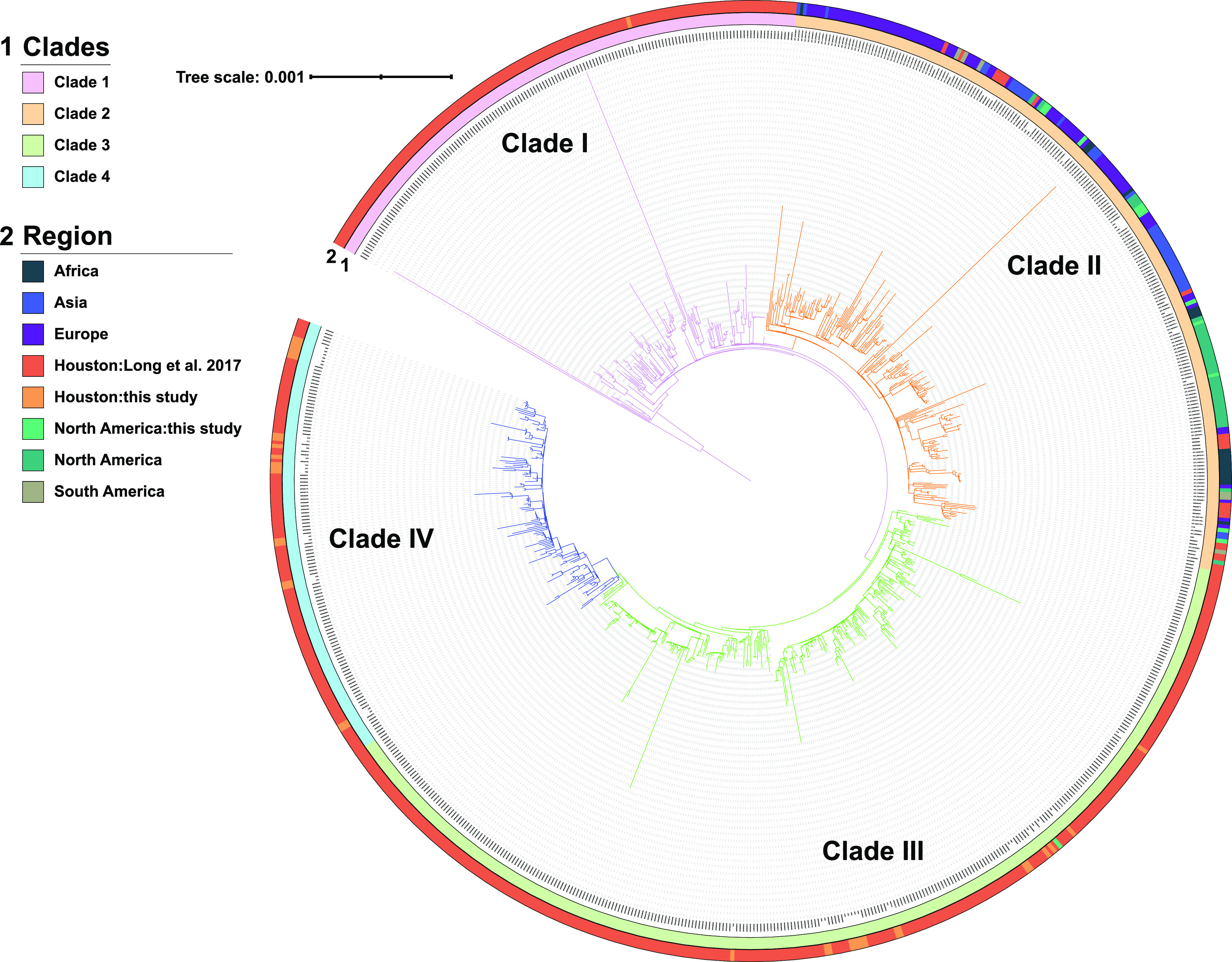
Population structure of previously characterized CG307 isolates with the CG307 Houston CRACKLE II isolates. Maximum-likelihood inferred phylogeny of CG307 isolates (*n* = 798) using C234 as a reference for core gene alignment with midpoint rooting. The branch label background corresponds to the hierBAPS predicted clade. The outer ring indicates the region where the isolate was collected. Clades I, III, and IV are predominantly made up of Houston isolates. Clade II is disseminated worldwide and shares a paraphyletic relationship with clade I.

To determine the overall population structure of CG307 isolates in the Houston region, relative to historical isolates detected globally, a reference-based alignment maximum-likelihood inferred phylogeny was generated with CG307 isolates (*n* = 798), using C234 (the first CG307 isolate in the Houston population), as a reference (Fig. 3). Four hierarchical population structures were detected, with a prominent distinction between three Houston-based ST307 clades (clades I, III, and IV) and the worldwide disseminated CG307 clade (clade II) (Fig. 3). Houston-based clade III isolates shared a common ancestor with clade IV isolates not shared with the distinct clade I and clade II isolates. Thus, phylogenetic reconstruction suggests that three lineages of CG307 (clades I, III, and IV) distinct from CG307 found in other parts of the world (clade II) are currently circulating in Houston hospitals.

### Dissection of the accessory genome suggests independent evolution and limited horizontal gene transfer between CG307 and CG258 lineages.

**FIG 4:**
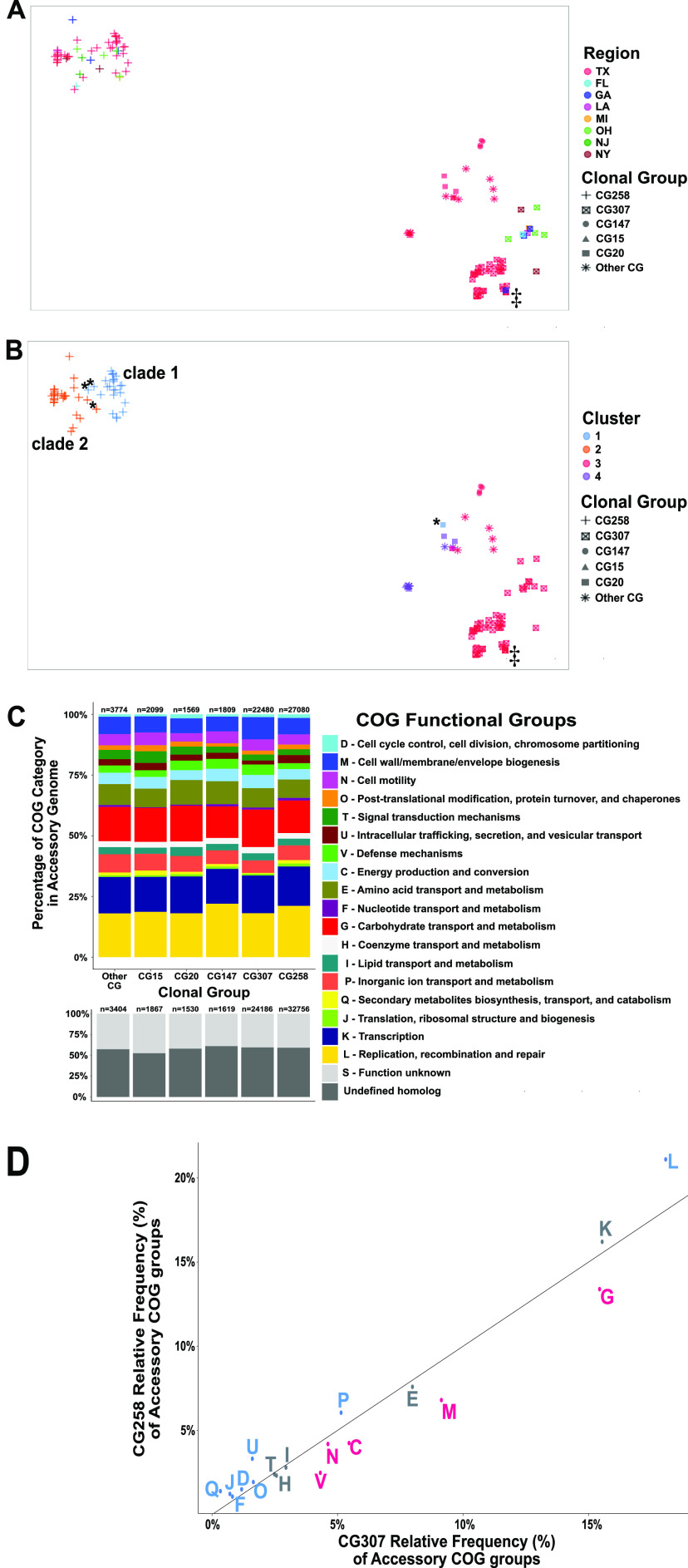
Clustering of CRKP isolates with predicted protein functional characterization of the accessory genome. (A and B) t-Distributed stochastic neighbor embedding (t-SNE) two-dimensional (2-D) plot of accessory genome clustering of CR*Kp* isolates. Clonal group is indicated by shape. ‡, Georgia isolate (C682) that clusters with Houston CG307 isolates. (A) Geographical region stratification of isolates. (B) Cluster group prediction (k = 4) using a PAM algorithm to determine cluster assignment. *, Exceptions to CG258 cluster-to-clade correlation. (C) Stacked bar chart of Cluster of Orthologous Genes (COGs) functional category proportions based on annotated genes found in the accessory genome. The *n* above each group indicates the absolute count of COGs identified in each clonal group per each genome. (D) The proportion of functionally annotated accessory genes differs significantly across pairwise comparisons of clonal groups as demonstrated in the scatterplot, which plots CG258 versus CG307 relative frequency proportions of COG functional groups denoted by the COG group letter (defined in panel C), with significant adjusted *P* values (Fisher’s exact test) indicated for a greater proportion of CG258 (blue) or CG307 (red) for each group labeled accordingly. These COG function group proportions exclude “S – unknown function” and “undefined homologs.” Nonsignificant COG functional group differences are labeled in gray.

We sought to determine the extent of accessory genome sharing within our CR*Kp* group as a measure of potential interclade horizontal gene transfer of antimicrobial resistance and virulence determinants. We took this approach to understand the dynamics of circulation of these genes between high-risk clones that might explain how they cocirculate in the Houston area. Thus, using the full cohort described in the previous section, we sought to determine the geographical clustering of the accessory genomes of all K. pneumoniae isolates, including genes encoding carbapenemases. Interestingly, both t-distributed stochastic neighbor embedding (t-SNE) analysis and principal-component analysis (PCA) indicated a distinct separation of the CG258 accessory genome from the rest of the isolates (Fig. 4A and B; Fig. S3 at https://gitlab.com/carmig_dissertations/shropshire_dissertation/crkp_supplemental_files), suggesting that CG258 has limited accessory genome sharing with other lineages. Fig. 4A shows that, apart from one Georgia isolate (C4688), CG307 isolates from Houston cluster together and are distinct from non-Houston CG307. In contrast to the unique accessory genome components in CG307 that segregated by region, we were unable to identify accessory genome clustering by geographical location of CG258 isolates.

We then identified the minimized genomic distance between each isolate to determine which isolates were more likely to share similar accessory genome content based on their predicted cluster assignment. Fig. 4B shows that when cluster groups were identified by sequence similarity (pairwise binary distances between the 121 isolates), there was a split in CG258 that largely segregated by *cps-*associated clades (cluster 1 versus cluster 2), with four exceptions (C293, C295, C259, and C4688). CG307 isolates shared a cluster assignment (cluster 3, Fig. 4B) with 10 other isolates belonging to non-CG307. Furthermore, CG307 accessory genomes appeared to cluster with CG147 isolates in our cohort. PCA of the accessory genome indicated that 90.7% of the variance in the data set could be explained in the first two components of the accessory genome with noted separation of CG258 from CG307, as well as the other clonal groups (Fig. S3 at https://gitlab.com/carmig_dissertations/shropshire_dissertation/crkp_supplemental_files). The PCA analyses in conjunction with t-SNE overall supports a clear separation between CG258 and non-CG258 accessory gene content.

To further support the divergence of CG258 from CG307 and the lack of genomic sharing, we analyzed a subset of the accessory genome (*n* = 2,943) excluding low- (≤5%) and high-frequency (≥95%) gene groups that are less indicative of horizontal gene transfer within the full cohort and determined distribution differences by Cluster of Orthologous Genes (COGs) functional categories (Table S1 at https://gitlab.com/carmig_dissertations/shropshire_dissertation/crkp_supplemental_files). Figure 4C shows the overall distribution of accessory genome content annotated by functional group across all clonal groups found. When focusing on COG functional group proportions of CG258 versus CG307 isolates, we found statistically significant differences in relative frequency proportions of each respective COG functional group with all but 5 of the 18 characterized COG functional groups (Fig. 4D). In contrast, there were fewer statistically significant differences observed in the proportion of COG functional groups within the accessory genomes for both CG258 and CG307 compared to each respective other clonal group found in the cohort (Fig. S4).

The largest statistically significant differences in COG functional groups between CG258 and CG307 were in predicted carbohydrate metabolism and transport mechanisms (G), cell membrane structure/biosynthesis (M), and replication/recombination/repair (L) genes (Fig. 4D). The larger proportion of replication, recombination, and repair genes (L) in CG258 is likely due to a higher mean number of plasmids per genome observed in CG258 than in CG307 isolates. Noted differences include a previously described chromosomal, 13-kb π-fimbriae gene cluster (12), conserved in all CG307 isolates (*n* = 48) and absent from all CG258 (*n* = 51) (adj. *P* value = 8e-33) (Fig. S5 at https://gitlab.com/carmig_dissertations/shropshire_dissertation/crkp_supplemental_files). The fimbrial gene cluster was also present in all four CG147 isolates. A second, previously described (12), chromosomal capsular synthesis cluster with 12 genes (Cp2) was present in all 48 CG307 isolates and absent from all others (adj. *P* value = 9e-34) (Fig. S5). Conversely, there was a carbohydrate metabolism operon (designated *ydjEFGHIJ*) exclusively found on all CG258 and CG147 isolates and absent in the CG307 isolates (adjusted *P* = 5e-34). Carriage of unique phages between CG307 and CG258 was a primary driver of accessory genome differences. Indeed, three intact phages detected in CG307 were absent in CG258 (Fig. S5).

We further subset the accessory genome based on the histogram of gene sharing (Fig. S6 at https://gitlab.com/carmig_dissertations/shropshire_dissertation/crkp_supplemental_files) to accessory genes shared in less than 80% of the population to observe gene sharing within and between each respective clonal group that does not include any potential “soft-core” genes. The median accessory gene groups shared within CG258 strains (640 [IQR: 557, 723]) was significantly higher than the number of gene groups shared between CG307 and non-CG258 strains (Fig. S7A). Similarly, a conservation of intragroup accessory genome sharing was observed within the CG307 strains (485 [IQR: 420, 550]) relative to intergroup accessory genome sharing with non-CG307 strains (Fig. S7B). Taken together, these results collectively indicate that CG258 and CG307 each contain highly conserved and distinct accessory genomes, which are likely driving independent endemic spread of each lineage in the Houston region.

### Carriage of chromosomal *bla*_CTX-M-15_ is the major antimicrobial resistance determinant of CG307 in the Houston area with evidence of independent acquisitions of *bla_KPC-2_*.

Table 3 provides an overview of the antimicrobial resistance profile of the CR*Kp* Houston isolates. The number of isolates carrying a carbapenemase gene was 84/95 (88.4%). The majority of the isolates harboring carbapenemase genes were *bla*_KPC-2_ carriers (77/95; 81.1%), followed by *bla*_KPC-3_ (4/95; 4.2%), *bla*_NDM-1_ (2/95; 2.1%), and *bla*_OXA-232_ (1/95; 1.1%). Most *bla*_KPC_ carriage occurred on the Tn*4401a* isoform (94%; Table S2 at https://gitlab.com/carmig_dissertations/shropshire_dissertation/crkp_supplemental_files). CG258 isolates harbored either *bla*_KPC-2_ (35/37; 94.6%) or *bla*_KPC-3_ (2/37; 5.41%), with the majority on pKpQIL plasmids (27/37; 73%), whereas most CG307 isolates (31/35; 88.6%) carried *bla*_KPC-2_ on different vectors, with one isolate (C678) carrying two *bla*_KPC-2_ copies, one on the chromosome and the other on a FIIK/FIB (pKpQIL) plasmid. The distribution of *bla*_KPC_-containing plasmids was more diverse in CG307, with the top three vectors responsible for CG307 Tn*4401a* transmission being FII(K)/FIB(K) plasmids (9/31, 29.0%), R plasmids (7/31, 22.6%), and the chromosome (6/31, 19.4%) (Fig. 1). Interestingly, six CG307 isolates harboring Tn*4401a* (the mean pairwise SNP difference is 40) had the same transposon insertion chromosomal context (Fig. 1) originating from five different Houston regional hospitals.

**TABLE 3:** Antimicrobial resistance features of 95 K. pneumoniae isolates from Houston, TX

Feature	Clonal Group:			*P* value^*b*^
	CG258 (*n* = 37) (%)^*a*^	CG307 (*n* = 35) (%)^*a*^	Other CG (*n* = 23) (%)^*a*^	
Carbapenemases				<0.0001
*bla*_KPC-2_	35 (94.6)	31 (88.6)	11 (47.8)	
*bla*_KPC-3_	2 (5.4)	0 (0.0)	2 (4.2)	
*bla*_NDM-1_	0 (0.0)	0 (0.0)	2 (4.2)	
*bla*_OXA-232_	0 (0.0)	0 (0.0)	1 (1.1)	
ESBLs				<0.0001
*bla*_CTX-M-15_	1 (2.7)	35 (100)	11 (47.8)	
*bla*_SHV-12_	30 (81.1)	0 (0.0)	0 (0.0)	
*bla*_SHV-27_	0 (0.0)	0 (0.0)	1 (4.3)	
*bla*_SHV-12_/*bla*_CTX-M-15_	1 (2.7)	0 (0.0)	0 (0.0)	
*ompK35/ompK36* changes^*c*^	37 (100)	35 (100)	5 (21.7)	<0.0001
Aminoglycoside^*d*^	36 (97.3)	30 (85.7)	15 (65.2)	0.003
Tetracycline^*d*^	2 (5.4)	3 (8.6)	9 (39.1)	0.002
Sulfonamide^*d*^	19 (51.4)	16 (45.7)	14 (60.9)	0.5
Phenicols^*d*^	14 (37.8)	8 (22.9)	9 (39.1)	0.3
Fluoroquinolone^*e*^	37 (100)	35 (100)	14 (60.9)	<0.0001
Colistin^*e*^	2 (5.4)	3 (8.6)	1 (4.3)	0.9

a Categorical responses include counts and percentages in parenthesis.

b Fisher’s exact test *P* value.

c Outer membrane porin gene changes are counted if a truncation or nonsense mutation is detected in either the *ompK35* or *ompK36* gene.

d Aminoglycoside, tetracycline, sulfonamide, and phenicol (e.g., chloramphenicol) predicted resistance based on the presence of at least one gene that confers resistance to the respective antibiotic class (e.g., *aac3-Ia* confers aminoglycoside resistance).

e Fluoroquinolone and colistin predicted resistance based on chromosomal mutations that confer resistance to each respective antibiotic class (e.g., truncations in *mgrB*/*pmrB* confer resistance to colistin). Small proportion of isolates (6/95; 6.3%) also carry quinolone resistance genes (i.e., *qnr* variants).

A characteristic feature of the Houston CG307 is that all isolates harbored more than one copy of *bla*_CTX-M-15_ (2 [*n* = 25], 3 [*n* = 9], and 4 [*n* = 1]) (Table 2 and Fig. 2B). *bla*_CTX-M-15_ was primarily located in the chromosome, with 2 to 3 copies per genome in relatively similar genomic contexts. Only 3 Houston CG307 isolates had plasmids (pC251_1, pC258_1, pC291_1) harboring *bla*_CTX-M-15_. All 3 plasmids were F-type pKPN3-like plasmids, which have been associated with CG307 isolates in other geographical locales (10, 11). In contrast to CG307, only 2/37 (5.4%) CG258 isolates harbored *bla*_CTX-M-15_, with the two isolates having the gene on distinct pKPN3-like plasmids (pC268_1 and pC608_2). The *bla*_SHV-12_ variant was the most common ESBL gene carried by CG258.

**FIG 5:**
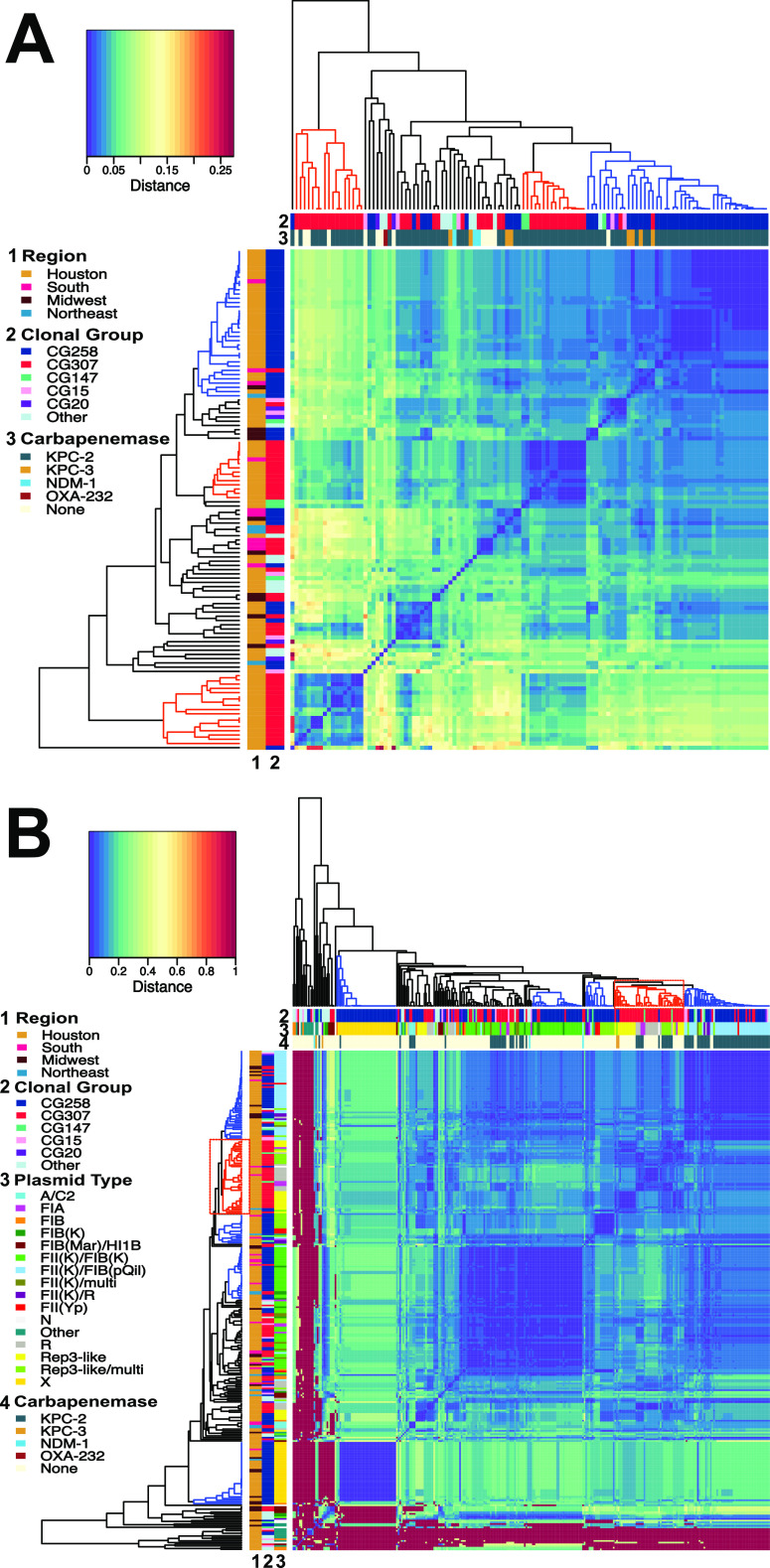
Dendrogram and heatmap of plasmidome mash distances. Legends for row and column labels are listed to the left of each graph. Each dendrogram is constructed through agglomerative hierarchical clustering using an “average” linkage. (A) Plasmidome mash distance matrix by isolate (*n* = 121). The Legend is labeled as follows: (1) region, (2) clonal group, (3) carbapenemase. There is a noted primary clustering group of CG258 isolates (blue-labeled clade), whereas there are two Houston-based CG307 clustering groups (red-labeled clades) with diffuse clustering occurring with other CGs. (B) Plasmidome mash distance matrix by plasmid type (*n* = 295) with small, primarily ColE1-like plasmids excluded from analysis. The legend is labeled as follows: (1) region, (2) clonal group, (3) plasmid type, (4) carbapenemase. This analysis shows clustering of primarily CG258 X2-type plasmids, multireplicon FIIK-type plasmids, and pKpQIL plasmids indicated with blue-labeled cluster groups. This is in contrast to one primary CG307 plasmid cluster group which includes R-type plasmids as well the novel pCG307_HTX plasmid associated with the Houston group.

Except for a CG307 Georgia isolate (C862) which shared similar genomic characteristics with CG307 from Houston, all other CRACKLE-2 CG307 recovered outside Houston exhibited distinct accessory genome features (Fig. 2B), supported by previous phylogenetic analyses (see above). Indeed, a majority of non-Houston CG307 (10/12, 83%) harbored a pKPN3-like plasmid, with 6 having *bla*_CTX-M-15_ as part of an IS*Ecp1* element. Furthermore, 3 of the 8 CG307 non-Houston isolates, which were *bla*_KPC_ positive, carried *bla*_KPC_ on non-Tn*4401a* transposons (compared to all Houston *bla*_KPC_-positive CG307 isolates, which carried *bla*_KPC_ on Tn*4401a*; Table S2 at https://gitlab.com/carmig_dissertations/shropshire_dissertation/crkp_supplemental_files). Additionally, non-Houston CG307s harbored A/C-type plasmids (*n* = 2) that carried *bla*_KPC_, a feature absent in Houston isolates (Fig. 5). These results, in conjunction with our phylogenetic analysis, support that a distinct epidemic lineage of CG307 is currently circulating in tertiary care hospitals in Houston, TX.

### CG258 and CG307 isolates harbor unique plasmid content.

To determine the full complement of plasmid vectors assembled in our CR*Kp* cohort, we characterized the full plasmid content (i.e., the plasmidome) to assess potential clustering and sharing of plasmids by clonal group. The plasmidome of our CR*Kp* isolates was highly diverse (Fig. 5A), with an average number of plasmid structures of 3.5 per isolate (minimum, 1; maximum, 7). Additionally, we analyzed the clustering of individual plasmids as the unit of analysis (Fig. 5B). Plasmid content clustered by core-defined clonal groups (Fig. 5A), with a majority of CG258 isolates clustering within their own distinct group (Fig. 5A, top right). In particular, there were three plasmid types associated with the CG258 lineage, including X3, FII(K)/FIB(K), and FII(K)/FIB (pKpQIL) plasmids (Fig. 5B).

There was a particular rep-3 family replication initator protein (52/415;12.5%) gene detected within plasmids found in nearly all Houston CG307 isolates (31/35; 88.8%). These CG307 plasmids associated with the aforementioned *repA* gene (here referred to as pCG307_HTX) primarily clustered with F-type and R-type plasmids (Fig. 5B, outlined with red dotted square). A total of 30/52 (57.7%) pCG307_HTX plasmids were predicted to be nonmobilizable, with the rest (22/52; 42.3%) predicted to be conjugative and/or mobilizable, harboring *tra* operons and/or *mob* encoding genes with essential *oriT* sites. Importantly, 10/31 (32.3%) pCG307_HTX plasmids carried Tn*4401a*-*bla*_KPC_ (Fig. 5B), suggesting that this plasmid may have been important for carbapenemase dissemination within the Houston CG307 isolates. Collectively, our results indicate that the CG258 plasmidome is conserved and largely segregated from other cocirculating CR*Kp* isolates, including CG307, in agreement with the pan-genome analyses.

### The novel pCG307_HTX plasmid carrying Tn*4401a* conjugates at similar rates compared to the CG258 pKpQIL plasmid.

To evaluate the potential for horizontal transfer of carbapenemase genes, we chose 4 plasmid candidates of interest based on their relative frequencies found in Houston CG307 (i.e., pCG307_HTX) isolates, non-Houston CG307 (i.e., pKPN3-307_typeA) isolates, and CG258 (i.e., pKpQIL) isolates. In particular, we assessed the relative conjugation transfer frequencies of two *bla*_KPC-2_-carrying plasmids associated with a rep-3 family *repA* gene (i.e., pCG307_HTX plasmids, one each predicted to be conjugative [pC299_2] or not conjugative [pC711_1], respectively). Conjugation efficiencies were compared using a comparable-size pKpQIL plasmid (pC344_1, CG258) that shared a similar *tra* operon and cargo gene region (Fig. S8 at https://gitlab.com/carmig_dissertations/shropshire_dissertation/crkp_supplemental_files). Additionally, we chose a pKPN3-307_typeA-like plasmid (pC763_2) primarily found in our non-Houston CG307 isolates to compare its conjugative frequency with the other aforementioned F-type plasmids (Fig. S8). Three of the four plasmids of interest had detectable, positive transconjugants in a proportion of conjugation transfer assay experiments (Fig. S9), with various efficiencies. pKpQIL (pC344_1) and pCG307_HTX (pC299_2) plasmids had comparable average transfer frequencies of 4.1 × 10^−7^ and 2.9 × 10^−6^, respectively. The pKPN_3_typeA plasmid-associated F-type plasmid had a greater transfer frequency (6.3 × 10^−5^). However, this did not reach a statistically significant difference (*P* = 0.06). As expected from *in silico* prediction, pC711_1 yielded no transconjugants in any of the three conjugation transfer assays. Overall, our conjugation experiments validated *in silico* predictions of mobilization and transmission of the novel pCG307_HTX plasmid associated with the Houston CG307.

## DISCUSSION

Although K. pneumoniae CG258 has been considered the major genetic lineage responsible for endemicity of carbapenem resistance, a new lineage designated K. pneumoniae CG307 has emerged in recent years (11–13). Since the first report of CG307 in the Netherlands in 2008, it has been identified in different parts of the world, including Colombia (23), Italy (24, 25), South Africa (26, 27), Pakistan (28), and South Korea (29), among others. Interestingly, the first detection of CG307 in the United States occurred in Houston, TX, in 2010, which also was to our knowledge, the first detection of CG258 in Houston (30) and was subsequently followed by an outbreak in a large, Houston hospital system (17). A more recent study assessing the clinical and genomic epidemiology of carbapenem-resistant *Enterobacterales* in the United States (3) showed that Houston was the first major city in the United States where carbapenem-resistant K. pneumoniae CG307 seemed to have established endemicity along with isolates belonging to CG258. Furthermore, a recent study suggests that CG307 may be spreading to other municipalities in South Texas and cocirculating with CG258 (31). The concomitant circulation of these major clones provided an opportunity to dissect their dynamics of dissemination, genetic relationships, and evolution using a pangenomic approach combined with clinical data. Our findings suggest that CG258 tends to have a higher median plasmid content, with a similar number of AMR determinants found across all CR*Kp* isolates.

The most striking finding of our study was that the two main multidrug-resistant K. pneumoniae lineages have evolved independently of one another and appear to be disseminating in parallel with limited evidence of interclade horizontal gene transfer between them. Moreover, our results support the notion than CG307 plasmids carrying KPC carbapenemases are likely to be shared with other clonal groups, except CG258, amplifying the epidemic of multidrug-resistant organisms in the same geographical area. Partitioning of the accessory genome by COG functional groups indicates large differences in distribution between CG258 and CG307 accessory genome content that are less apparent when comparing each respective clade with other clonal groups. These differences are potentially driven, in part, by CG307 having a greater proportion of carbohydrate metabolism and cell membrane synthesis determinants. Virulence factors found in our cohort, such as a separate chromosomal capsular synthesis locus (Cp2) shared across CG307 isolates and plasmid-borne glycogen synthesis clusters found on CG307 plasmids, have been previously documented in CG307 isolates found in Colombia and Italy (12).

Virulence factors such as the filamentous, extracellular organelles and type 1 fimbriae are associated with colonization and infection of the urinary tract in Escherichia coli and K. pneumoniae (32–35). Interestingly, a unique π-fimbria gene cluster (12) was strongly associated with CG307 and CG147 isolates, with urine as a main source where CG307 was recovered. Furthermore, we found accessory genome intersection between CG307 and CG147 isolates by clustering analysis, with comparable proportions of COG functional groups carried by each group. Indeed, there has been recent evidence of parallel antimicrobial resistance patterns shared between CG307 and CG147 with acquisition of similar F- and R-type plasmids harboring *bla*_CTX-M-15_ along with identical *gyrA* and *parC* mutations conferring quinolone resistance, suggesting a common evolutionary pathway (12).

Our study also identified a unique plasmid (designated pCG307_HTX) in predominantly Houston CG307 isolates carrying a rep-3 family initiator replication protein with potential for vertical and horizontal transmission based on predicted mobility, as well as positive transconjugants in our conjugative transfer assays. A similar rep-3 family plasmid that recombined with an FII(K) plasmid had been described in a CTX-M-15-associated ST416 K. pneumoniae isolate (pKDO1; GenBank accession no. JX424423) (36). We also found that the previously identified FII(K)/FIB(K) plasmid (12), harboring *bla*_CTX-M-15_ associated with worldwide CG307 isolates, had relatively higher conjugative transfer efficiency than the Houston pCG307_HTX and CG258 pKpQIL plasmids. This FII(K)/FIB(K) plasmid harboring *bla*_CTX-M-15_ was less prevalent in the Houston CG307 cohort (*n* = 3) where the primary vector of ESBL transmission was stable integration of two copies of a chromosomal IS*Ecp1*-*bla*_CTX-M-15_ transposon. We found that while CG258 may have a greater number of plasmids per genome, plasmidome analyses suggest a greater diversity of unique vectors carrying genes encoding carbapenemases for the CG307 lineage with the potential for sharing across other circulating non-CG258 CR*Kp* isolates in the Houston region. These genomic features (i.e., stable 2X *bla*_CTX-M-15_ copy chromosomal integration with a diversity of carbapenemase vectors) in conjunction with antimicrobial stewardship practices may be the primary drivers of the CG307 dissemination in Houston, TX.

Interestingly, the propensity for disseminating multiple AMR determinants to other clonal groups was noted in a recent outbreak of CG307 in northeast Germany (37). In contrast to our study, they had also found the potential of convergent hypervirulent and multidrug-resistant determinants in CG307 isolates due to promiscuous sharing of plasmids carrying multiple hypervirulent and AMR determinants (37). Our epidemiological investigation along with this northeast Germany surveillance study indicate the high potential of horizontal gene transfer along with noted regional variation in accessory genome content across different CG307 clades.

There are limitations in our study. Due to the nonrandom sampling conducted in our study, the extent to which CG307 is disseminated in other geographical locales could be much greater than what was inferred from our study. Nevertheless, since selection criterion were comparable across study sites, we believe direct comparisons can be made across CRACKLE-2 study sites. While we have extensive molecular epidemiological data, our limited traditional epidemiological data limit our ability to assess potential transmission between our participants and clonal outbreaks within our data set (e.g., we could not establish if a Georgia patient with a CG307 isolate that shared high genomic similarity to the CG307 Houston isolates had any epidemiological links with the Houston region.). Caution should be exercised when inferring differences in clinical outcomes of patients with CG258 versus CG307 due to inclusion of both infection and colonizing isolates with small sample sizes, especially when considering statistically significant differences in 30-day mortality between clonal groups. Additionally, due to small sample sizes, some comparisons may be underpowered (i.e., resistance classes, virulence genes, and Kleborate scores across clonal groups), and thus we may fail to detect some differences in AMR composition. Kleborate scores only incorporate a limited number of epidemiologically relevant K. pneumoniae features, which may limit direct comparison of virulence and AMR genetic factors. While we did not causally link the type 1 fimbriae associated with CG307 isolates with their higher prevalence found in urine versus CG258, future research should aim to explore potential tropism of CG307 in the urinary tract.

In conclusion, our data provide a detailed dissection of parallel epidemics of carbapenem-resistant K. pneumoniae high-risk lineages, both of which are considered urgent public health issues. The genomic and clinical insights presented here are likely to provide novel understanding of the genomic epidemiology of multidrug-resistant K. pneumoniae in order to improve CR*Kp* detection and surveillance.

## MATERIALS AND METHODS

### Study design.

Characterization of patients and isolates was based on the CRACKLE-2 study (3), a prospective, multicenter observational cohort study performed in 49 hospitals across the continental United States. This study focused on patients recruited in Houston, TX, and their isolates. A total of 160 CRE isolates were collected from 10 hospital sites within the Houston, TX, metropolitan area from April 2016 to December 2017. Clinical isolates were identified as belonging to the *Enterobacterales* group, and antimicrobial susceptibility testing was performed using Etest and Vitek platforms. Inclusion criteria have been described previously (3). A total of 95/160 CRE (59.4%) were identified as belonging to the K. pneumoniae species complex (KpSC) and were isolates subjected to whole-genome sequencing. Further details of the study design are in supplemental methods (at https://gitlab.com/carmig_dissertations/shropshire_dissertation/crkp_supplemental_files).

### Whole-genome sequencing.

Isolate culture, genomic DNA extraction, short-read sequencing library preparation, and Illumina short-read sequencing have been described previously (3). All isolates identified as CR*Kp* in our sampling frame were subjected to Oxford Nanopore Technologies (ONT) long-read sequencing using the SQK-RBK004 library preparation kit and sequenced on an Oxford Nanopore GridION X5 (Oxford, UK). The hybrid assembly pipeline has been described previously (38). Genome assembly metrics are shown in Table S4 (at https://gitlab.com/carmig_dissertations/shropshire_dissertation/crkp_supplemental_files) with their respective BioSample accession and Antibacterial Resistance Leadership Group (ARLG) identification numbers. A summary of the molecular epidemiological results found using Kleborate v1.0.0 (18) can be found in Table S5. A matrix of all *in silico* PCR-based replicon typing found with PlasmidFinder (39) is included in Table S6. A full list of RefSeq/SRA accession numbers and CRACKLE IDs for the CG307 phylogeny (*n* = 798) can be found in Table S7. A full description of WGS, genome assembly, and comparative genome analysis can be found in the supplemental methods.

### Pangenome analyses.

Panaroo v1.2.2 (40) was used to generate a pangenome profile from the 121 gff files generated from Prokka v1.14.5 (41) using the “sensitive” clean mode for gene contamination filtering. Three reference isolates were included, NJST258_1 (GenBank accession no. GCA_000598005.1), NJST258_2 (GenBank accession no. GCA_000597905.1), and KPN11 (GenBank accession no. GCA_002148835.1). Isolate C719 was not included in the pan-genome analysis since it is a member of the *K. quasipneumoniae* subsp. *quasipneumoniae* (Kp2) phylogroup (42). Further details regarding our pan-genome analysis, plasmidome analysis, and phylogenetic as well as clustering analysis can be found in the supplemental methods.

### Conjugation transfer assays.

Four isolates with plasmids of interest based on plasmidome analysis were included in conjugation transfer assays using a modified protocol (43). Details of the plasmids studied can be found in Table S8 (at https://gitlab.com/carmig_dissertations/shropshire_dissertation/crkp_supplemental_files). Easyfig was used to compare plasmid structures (44). Donors and a sodium azide (NaN_3_)-resistant E. coli strain, J53, were grown overnight in Trypticase soy broth (TSB) supplemented with 2 μg/mL ertapenem or 10 μg/mL gentamicin for plasmid selection and 150 μg/mL NaN_3_ for counterselection at 37°C with mild agitation. Overnight cultures were washed in 0.9% NaCl twice and then subcultured into fresh TSB at a 1:100 dilution and incubated at 37°C until the mid-log phase (∼0.6 optical density at 600 nm [OD_600_]). Broth mating was performed with 1:10 donor-to-recipient ratios with TSB overnight at 37°C for 20 h. Trypticase soy agar (TSA) plates supplemented with 2 μg/mL ertapenem or 10 μg/mL gentamicin and 150 μg/mL NaN_3_ were used to select for transconjugants. Conjugation transfer frequency was enumerated by calculating the ratio of CFU/mL in transconjugants over the CFU/mL in the donor plates. The limit of detection was calculated by taking the minimum CFU threshold detected factoring in the dilution factor (50 CFU/mL) and dividing that by donor frequency. We used a PCR protocol to check for the positive identification of transconjugants screening for *bla*_KPC-2_, *aacA4*, E. coli J53 (*rpoB* gene), as well as plasmids of interest with the primers listed in Table S9.

### Ethics approval and consent to participate.

Original data were collected with the approval of The University of Texas Health Science Center at Houston Institutional Review Board (IRB), the Committee for Protection of Human Subjects (CPHS; protocol ID HSC-MS-16-0334; ethical approval 5/16/2016), and no further data were collected from these subjects. CRACKLE-2 data collected outside Houston was approved through the Duke University Health System Institutional Review Board for Clinical Investigations (DUHS IRB; protocol ID Pro00071149; ethical approval 6/29/2016) with no clinical data reported in this study. All clinical isolates were stripped of identifying information prior to analysis. All clinical data were deidentified upon receipt.

### Statistics.

Group-level distributions of continuous variables were evaluated using a one-way analysis of variance (ANOVA) test or Kruskal-Wallis rank-sum tests contingent on assumptions based on the data distributions. *Post hoc* tests for continuous variables with significant ANOVA *P* values were accomplished using Tukey’s honest significance test or the Dunn test using the “FDR” method to control for multiple comparisons. The Wilcoxon rank-sum test was used to determine distribution differences of continuous variables across two groups. Distributions of categorical data were evaluated using the Pearson chi square or Fisher’s exact test, dependent on cell counts. All statistical analysis was performed with R v4.0.0 software.

### Data availability.

Short-read and long-read FASTQ files along with complete assemblies for all K. pneumoniae isolates sequenced, including isolates previously published (3), are in BioProject PRJNA658369. The supplemental data sets, figures, and methods supporting the conclusions of this article are included within Supplemental material including scripts, tables, figures, and methods are publicly available through the FigShare open access repository, https://doi.org/10.6084/m9.figshare.19411718.v1.
